# Comparing maximum rate and sustainability of pacing by mechanical vs. electrical stimulation in the Langendorff-perfused rabbit heart

**DOI:** 10.1093/europace/euw354

**Published:** 2016-12-23

**Authors:** T. Alexander Quinn, Peter Kohl

**Affiliations:** 1 Department of Physiology and Biophysics, Dalhousie University, 5850 College St, Halifax, NS B3H 4R2, Canada; 2 Institute for Experimental Cardiovascular Medicine, University Heart Centre Freiburg/Bad Krozingen, Medical School of the University of Freiburg, Elsaesser Str 2Q, 79110 Freiburg, Germany; 3 National Heart and Lung Institute, Imperial College London, The Magdi Yacoub Institute, Hill End Road, UB9 6JH London, UK

**Keywords:** Cardiac, Electrophysiology, Mechano-electric feedback, Optical mapping, Stretch-activated channels, Strain

## Abstract

**Aims:**

Mechanical stimulation (MS) represents a readily available, non-invasive means of pacing the asystolic or bradycardic heart in patients, but benefits of MS at higher heart rates are unclear. Our aim was to assess the maximum rate and sustainability of excitation by MS vs. electrical stimulation (ES) in the isolated heart under normal physiological conditions.

**Methods and results:**

Trains of local MS or ES at rates exceeding intrinsic sinus rhythm (overdrive pacing; lowest pacing rates 2.5±0.5 Hz) were applied to the same mid-left ventricular free-wall site on the epicardium of Langendorff-perfused rabbit hearts. Stimulation rates were progressively increased, with a recovery period of normal sinus rhythm between each stimulation period. Trains of MS caused repeated focal ventricular excitation from the site of stimulation. The maximum rate at which MS achieved 1:1 capture was lower than during ES (4.2±0.2 vs. 5.9±0.2 Hz, respectively). At all overdrive pacing rates for which repetitive MS was possible, 1:1 capture was reversibly lost after a finite number of cycles, even though same-site capture by ES remained possible. The number of MS cycles until loss of capture decreased with rising stimulation rate. If interspersed with ES, the number of MS to failure of capture was lower than for MS only.

**Conclusion:**

In this study, we demonstrate that the maximum pacing rate at which MS can be sustained is lower than that for same-site ES in isolated heart, and that, in contrast to ES, the sustainability of successful 1:1 capture by MS is limited. The mechanism(s) of differences in MS vs. ES pacing ability, potentially important for emergency heart rhythm management, are currently unknown, thus warranting further investigation.

What’s new?

- Localised non-contusional mechanical stimulation can pace the ventricles in isolated whole-heart.
- Maximum pacing rates achievable with mechanical stimulation are lower than with electrical stimulation.
- Pacing capture by mechanical stimulation at rates exceeding normal sinus rhythm is reversibly lost after a finite number of cycles; this number decreases with increasing pacing rate, even though the stimulated tissue remains electrically excitable.
- When interspersed with electrical stimuli, the number of mechanically-paced beats to loss of capture is lower than with same-frequency mechanical stimulation only.
- This suggests the presence of a ‘depletable pool of mediator(s)’ required for mechano-electric coupling, which is also sensitive to electrical stimulation.
- The nature of the ‘mediator(s)’ is currently unknown.

## Introduction

While implanted electrical pacemakers are an effective means of sustained cardiac pacing, mechanical pacing is of interest for the emergency resuscitation setting, as it represents a rapidly available, non-invasive and generally well-tolerated method for triggering cardiac excitation in the asystolic or severely bradycardic heart.^1^

Localized mechanical stimulation (MS) of the human heart, whether by direct tissue contact of intra-cardiac devices (catheters, pacing leads)^2^ or by extracorporeal impact (*Commotio cordis*,^3^ precordial thump^4^) can cause mechanically-induced ventricular excitation (VE_M_), resulting in competent ventricular contraction. If applied rhythmically to the precordium (‘precordial percussion’),^5^ repetitive thumps have been shown to be an effective means for extracorporeal pacing of the asystolic^6^^,^^7^ or bradycardic^8^ heart, while benefits of MS for cardioversion of tachycardias is limited.^9^ As mechanically-induced heartbeats have a greater hemodynamic effect than external chest compressions,^10^ they can maintain consciousness in patients during extended periods of ventricular standstill (cases as long as 2 h 45 min have been reported).^6^^,^^11^

This ‘mechano-electric feedback’ effect^12^ was exploited by resuscitation pioneer Paul Zoll in the design of a mechanical pacing device for external stimulation of heart beats in emergency settings (‘cardiac thumper’).^13^ In dogs with normal sinus rhythm and high-degree atrioventricular block, repetitive heartbeats were evoked using this device, with no cases of mechanically-induced sustained arrhythmia (such as tachycardia or fibrillation, which can occur following VE_M_^14^), even when stimulation occurred during the relative refractory period and at energies up to 10 times VE_M_ threshold. The device was also shown to be effective in patients with asystole after ventricular fibrillation, with atrial fibrillation, or with implanted pacemakers for atrioventricular block.^13^

More recent device-based mechanical pacing efforts have focused on the use of extracorporeal high intensity focused ultrasound,^15^ which has been shown to excite frog,^16^ mouse,^17^ rat,^18^ and pig^19^ hearts, and intravenously injected magnetic microparticles manipulated by an external electromagnet, which can mechanically pace the right ventricle of rat and pig.^20^ Maximum rates and sustainability of repetitive mechanical pacing, however, have remained ill-explored.

Using optical mapping of direct local MS of the ventricular epicardium in rat^21^ and rabbit^22^ isolated hearts, we have shown that VE_M_ originates focally from the stimulation site, spreading downstream from the point of earliest activation in a manner that is indistinguishable from electrically paced beats (including parameters such as d*V*_m_/d*t*_max_, action potential duration, conduction velocity). Mechanical pacing occurs by activation of cation-non-selective stretch-activated channels (SAC_NS_),^23^ as it is prevented by SAC_NS_ block with *Grammostola spatulata* MechanoToxin-4 (GsMTx-4).^22^ In terms of macroscopic mechanics, VE_M_ depends on the extent of local tissue indentation.^22^ Whether repetitive supra-threshold mechanical stimuli can continuously cause excitation in the spontaneously-active isolated heart, and if so, at what maximum rate, is unknown. The goal of this study, therefore, was to investigate the maximum rate and sustainability of local VE_M_ induction, compared to same-site electrical stimulation (ES), in the isolated Langendorff-perfused rabbit heart.

## Methods

### Ethical approval

This study was carried out, with local ethical approval, in accordance with the UK Home Office *Animals* (*Scientific Procedures*) *Act* of 1986. Details of experimental protocols have been reported following the Minimum Information about a Cardiac Electrophysiology Experiment (MICEE) reporting standard,^24^ see online repository (https://www.micee.org/?q=node/00001378).

### Heart preparation

Female New Zealand White rabbits (1.4 ± 0.3 kg) were euthanized by ear vein injection of 140 mg/kg pentobarbital. After thoracotomy, the heart was swiftly excised and placed in Krebs–Henseleit solution (containing [in mM]: 120 NaCI; 4.7 KCl; 24 NaHCO_3_; 1.4 NaH_2_PO_4_; 1.0 MgCl_2_, 1.8 CaCl_2_; 10 glucose; osmolality: 300 ± 5 mOsm/kg; pH: 7.4 ± 0.05) bubbled with carbogen (95% O_2_, 5% CO_2_). The heart was connected rapidly (1–2 min from excision) and bubble-free to a custom Langendorff apparatus by aortic cannulation, and perfused with 37°C Krebs–Henseleit solution at 15 mL/min. Perfusion pressure was monitored with a transducer (TSD104A; Biopac Systems Inc., Goleta, CA), and temperature with a fast-response thermistor (TSD202A; Biopac Systems Inc.) positioned in the aortic cannula via a three-way stopcock. An incision into the proximal pulmonary artery allowed coronary effluent to exit the right ventricle. Remaining extra-cardiac tissue (lungs, thymus, pericardium, vessels) was removed. For mechanical support during epicardial MS, the heart was positioned into an individually pre-molded Parafilm (Bemis Company Inc., Oshkosh, WI) cradle with black backing, and the entire perfusion system was angled at 45°, with the left ventricle (LV) facing upwards, to allow surface-perpendicular mechanical contact during optical measurements. The exposed epicardial surface was superfused with warm Krebs–Henseleit solution at a rate of 1 mL/min.

**Figure 1:**
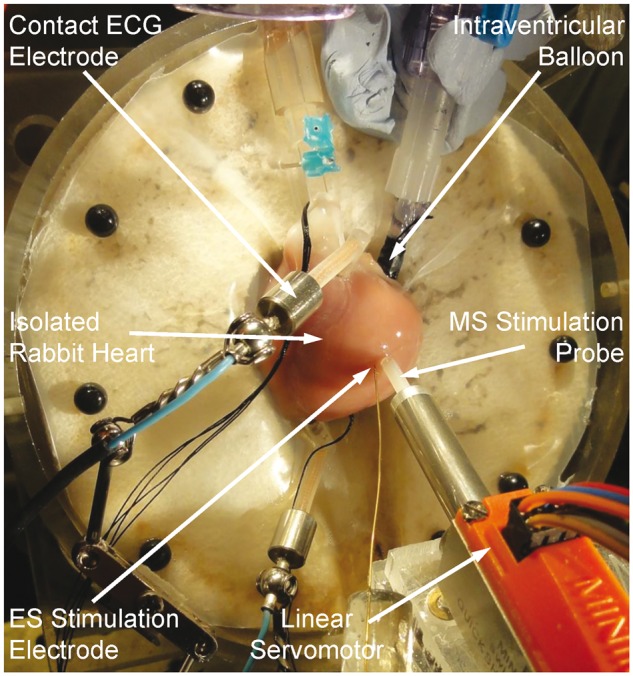
Photographic image of the experimental setup, showing an isolated rabbit heart instrumented with contact electrocardiogram (ECG) electrodes, an intraventricular balloon, electrical stimulation (ES) electrode and mechanical stimulation (MS) probe, coupled to a linear servomotor.

A 4–5 mm incision was made in the mid-left atrial auriculum. A short piece of 18 G intravenous cannula was passed through the incision, across the mitral orifice into the LV and pushed transmurally though the apex to prevent intra-ventricular fluid build-up. A custom-made pre-strained deflated polyethylene balloon, fitted on a 10 mm piece of manometer line (2 mm inner diameter) filled with degassed water and connected to a three-way stopcock, was inserted into the LV via the auricular incision. The balloon tip was secured at the ventricular apex by a 3–0 silk suture through the apical cannula. The atrium was tied to the manometer line by a silk ligature to secure the base of the balloon inside the LV. Intra-ventricular pressure was monitored with a transducer (TSD104A; Biopac Systems Inc.) connected to the balloon stopcock. The balloon, pressure transducer and connections were kept air-free to prevent damping of the pressure signal. A surface ECG was measured using two spring-loaded monopolar Ag/AgCl pellet electrodes (PY2 73-0200; Harvard Apparatus, Holliston, MA), one contacting the right atrium and the other the LV apex. Temperature, perfusion pressure and ECG sensors were interfaced with a data acquisition system (MP150; Biopac Systems Inc.) and data were collected at 2 kHz. After a 15 min equilibration period, the intra-ventricular balloon was inflated with degassed water to a diastolic pressure of 0–5 mmHg. The experimental setup can be seen in *Figure 1*.

### Mechanical and electrical stimulation

Comparison of MS and ES was performed on *n *=* *6 spontaneously active healthy hearts. Local MS was applied at the mid-LV free-wall by epicardial indentation with a 3.1 mm^2^ contact-area probe. This was accomplished by swift forward and reverse motion of the probe by a computer-controlled linear DC-servomotor (LM 1247-02-01; Faulhaber MiniMotor SA, Croglio, Switzerland) regulated by a motion controller with position decoder (MCLM 2006 S; Faulhaber MiniMotor SA) using custom programs developed in Motion Manager (Faulhaber MiniMotor SA). In an initial series of additional hearts (*n *=* *3), the degree of tissue indentation needed to ensure reliable induction of excitation was assessed. In agreement with previous results (in *n *=* *7 separate hearts), the threshold for VE_M_ (at an indentation rate of 300 mm/s) was ∼2 mm indentation.^22^ Thus to ensure reliable induction of VE_M_, a 3 mm indentation depth was chosen for MS (1.5× threshold), with indentation and retraction each occurring over 10 ms.

Local ES (2 ms bipolar pulse, with voltage set to 1.5× threshold, generally ∼3 V) was applied at the center of the same mid-LV free-wall location using a point (100 μm diameter) concentric bipolar stimulation electrode (SNE-100; Lohmann Research, Castrop-Rauxel, Germany).

Trains of 200 MS or ES were applied at stimulation rates that increased from 0.5 Hz above intrinsic sinus rate to 6.5 Hz, in 0.5 Hz increments, with a 1 min recovery period of normal sinus rhythm between each stimulation period. Timing relative to the cardiac cycle of the first stimulus in each train was controlled by triggering from the peak of the ECG R-wave to be diastolic using custom-designed electronics and programs developed in MATLAB (MathWorks, Natick, MA).

To evaluate the focal nature of excitation, following the MS/ES train series, hearts were loaded with a voltage-sensitive fluorescent dye (20 μL bolus of 27.3 mM di-4-ANBDQPQ solution in medical grade ethanol injected directly into the aortic cannula in 0.4 μL increments over 2 min, i.e. diluted in 30 mL of perfusate to an effective concentration of 23.4 μM; dye acquired from the University of Connecticut Health Center, Farmington, CT). Optical mapping was performed as described previously.^21^ Briefly, fluorescence was excited by two red light-emitting diodes (CBT-90-R; Luminus Devices Inc., Billerica, MA) through band-pass filters (D640/20X; Chroma Technology Corp., Bellows Falls, VT). Emission was collected with a 50 mm high-speed lens (DO-5095; Navitar, Rochester, NY) through a long-pass filter (HQ690LP Chroma Technology Corp.) and recorded at 511 frames per second by a 128 × 128 pixel, 16-bit electron multiplying charge coupled device camera (Cascade: 128+; Photometrics, Tucson, AZ) controlled using MultiRecorder (developed by Stefan Luther and Johannes Schröder-Schetelig, Max Planck Institute for Dynamics and Self-Organization, Göttingen, Germany).

To evaluate whether MS was indeed sub-contusional, tissue integrity was assessed by analysis of creatine kinase activity in coronary effluent (17296H CK-NAC Liquid; Alpha Laboratories Ltd., Eastleigh, United Kingdom) using a spectrophotometer (BioTech UV1101; Biochrom WPA, Cambridge, United Kingdom), which has been previously shown to reliably track tissue damage associated with contusional MS in rabbit isolated hearts.^25^

### Data analysis

Data were analyzed using custom programs in Matlab (MathWorks). Values are presented as mean ± standard error, with means compared by two-tailed, paired Student’s *t*-test, with a *P* value *< *0.05 indicating a statistically significant difference between means.

## Results

**Figure 2:**
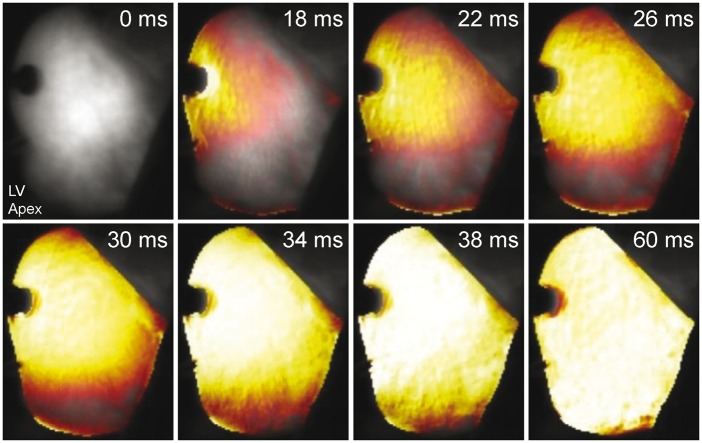
Mechanically-induced focal excitation of the left ventricle visualised by epicardial optical mapping (frames taken from Movie S1; see Supplementary material online).

**Figure 3:**
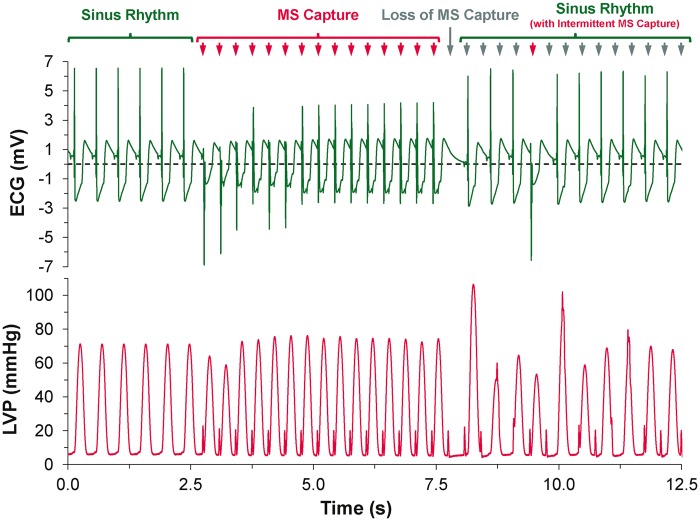
Electrocardiogram (ECG) and left ventricular pressure (LVP) recordings during sinus rhythm, followed by a train of mechanical stimulations (MS) at a rate of 3 Hz during which loss of 1:1 capture occurs, resulting in a return to sinus rhythm with intermittent MS capture.

**Figure 4:**
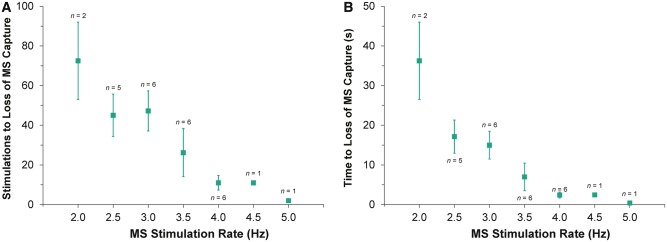
Effect of overdrive pacing rate by mechanical stimulation (MS) on the number of stimulations (*A*) and time (*B*) to loss of MS 1:1 capture.

Supra-threshold MS reliably caused focal excitation from the site of stimulation (*Figure 2*, see Supplementary material online, *Movie S1*). With repetitive stimulation at rates exceeding spontaneous pacemaker activity (overdrive pacing), the maximum rate of stimulation at which 1:1 capture occurred was lower for MS than for ES in all hearts (4.2 ± 0.2 vs. 5.9 ± 0.2 Hz; *P* = 0.001). For each overdrive pacing rate with 1:1 MS capture (i.e. at stimulation rates between 2.5 ± 0.5 Hz and 4.5 ± 0.5 Hz), ES was sustained for the entire 200-cycle stimulation period. With MS, however, capture was lost during the period of stimulation (*Figure 3*), after a finite number of stimuli. The number of consecutive MS with 1:1 capture, and the duration of 1:1 capture, decreased with increasing stimulation rate (*Figure 4*). The loss of capture with MS was reversible, as VE_M_ re-occurred after a 1 min recovery period of spontaneous sinus rhythm. During MS failure, there was no general loss of LV excitability, as ES applied to the same LV tissue site was still able to excite the heart.

There was no tissue damage, as confirmed by a lack of creatine kinase increase in the coronary effluent, the absence of changes in either heart rate or ECG configuration during return to normal sinus rhythm, and the ability to resume same-site MS after a 1 min pause.

**Figure 5:**
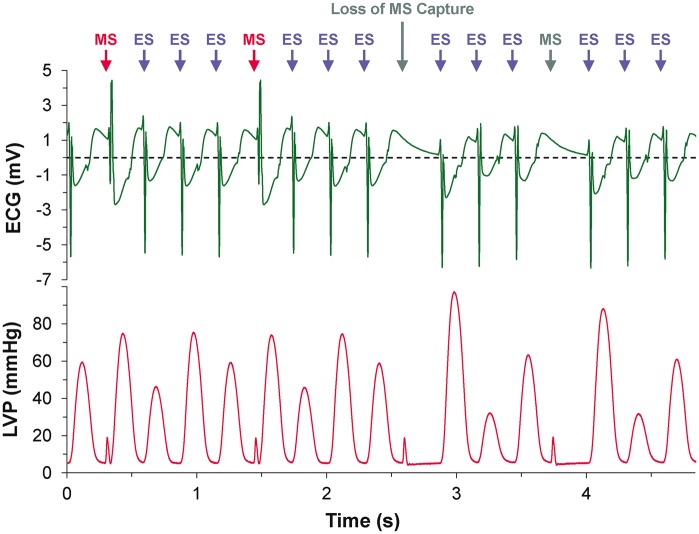
Electrocardiogram (ECG) and left ventricular pressure (LVP) recordings during a train of 1:3 mechanical:electrical (MS:ES) stimulations at a total stimulation rate of 3 Hz during which loss of MS capture occurs after 2.5 s, while ES capture is maintained.

**Figure 6:**
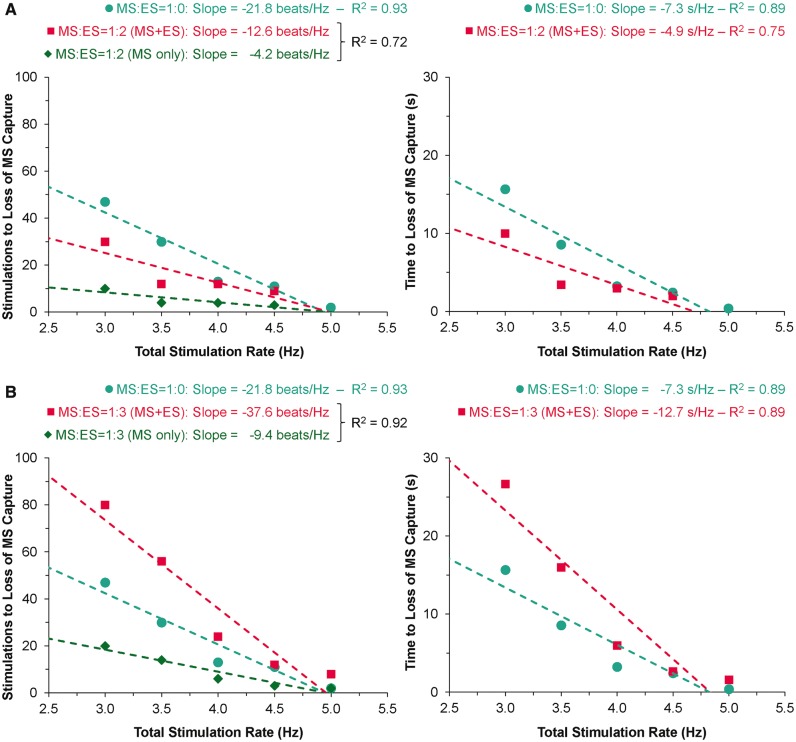
Effect of total stimulation rate on the number of total (mechanical + electrical, MS + ES) and MS-only stimulations (left) and time (right) to loss of MS 1:1 capture for trains of MS only (1:0) and mixed MS:ES at ratios of 1:2 (*A*) or 1:3 (*B*). Note that the green diamonds show the number of MS during MS:ES trains (net rate of MS is reduced by interspersed ES). The dashed lines were generated by linear regression.

To further investigate the relation of mechanical and electrical capture, series of MS were interspersed with ES (thus reducing the effective MS application rate, while maintaining total pacing rate; *Figure 5*). Counterintuitively, at MS:ES ratios of 1:1 and 1:2, there was a decrease in the total number of stimulations (combined MS and ES) and time to loss of capture by MS (*Figure 6A*). At further decreased relative contributions of MS (MS:ES ratios of 1:3 or more), the total number of stimulations (MS and ES combined) and time to loss of capture by MS were increased (*Figure 6B*). However, for all MS:ES trains, the number of MS that could be applied before a loss of capture occurred was reduced by comparison with trains consisting of MS only (green diamonds vs. blue circles in *Figure 6*). Capture by ES was maintained throughout all protocols (*Figure 5*).

## Discussion

This study sought to compare maximum rates and sustainability of MS vs. ES in isolated Langendorff-perfused rabbit hearts. Repetitive local MS of the LV epicardium causes repeated focal VE_M_. The maximum rate at which stimulation is able to achieve 1:1 capture is lower for MS than for ES. At all overdrive pacing rates for which repetitive MS is possible (roughly 2–5 Hz), 1:1 capture is reversibly lost after a finite number of stimulations, which may account for the loss of capture seen with continuous focused ultrasound^15^ and magnetic microparticle-based pacing.^20^ At the same time, the tissue remains electrically excitable, as capture by ES is maintained. The number of MS that can be applied until loss of capture decreases with rising stimulation rate. Surprisingly, if interspersed with ES, the maximum number of MS to failure of capture is lower than for same-frequency MS-only pacing.

### Potential mechanisms

The findings that MS and ES allow different maximum pacing rates and that upon loss of MS capture excitation is still possible by ES suggest that MS and ES are subject to different types of (mechanical and/or electrical) ‘refractoriness’. This is supported by a study of repetitive local stimulation of LV epicardium in open-chest anesthetised dogs that demonstrated a decrease in the effective refractory period at the site of stimulation with ES (at a pacing rate of 2.5 Hz), but showed no change with MS, which could bias maximum achievable pacing rates in favour of ES.^26^ In a study employing transient inflation of an intra-ventricular balloon for MS (which has been described as an effective means for pacing the asystolic heart),^27^ repeat inflations were effective only after periods of rest (up to 1 min for full recovery of mechanically-induced excitability).^28^ Overall, these studies suggest that MS involves a ‘depletable yet replenishable’ pool of mechano-electric ‘mediator(s)’ that is different from established electrical mechanisms of refractoriness.

Potential mechanisms for a mechano-electric adaptation period, during which there is a temporary reduction of VE_M_-inducibility that returns after a period of normal sinus rhythm (demonstrated in this and previous reports), as well as the difference in maximum stimulation rates for MS and ES (shown here), include MS-specific effects on: (i) tissue mechanical properties (passive or viscoelastic); (ii) cytoskeletal elements; (iii) SAC_NS_ (or other ion channel activity); (iv) ionic distributions and/or availability; (v) intracellular (such as sarcoplasmic reticulum, mitochondria or lysosomes) or sarcolemmal domains (e.g. caveolae); (vi) second messenger systems that influence the above; or (vii) other unknown factors necessary for MS.

In theory, effects of repetitive mechanical stimulation on the mechanical properties of cardiac tissue could be an important mechanism involved in the use-dependent loss of MS-related excitability. For instance, during mechanical testing of passive whole hearts, ‘strain softening’ has been reported, by which tissue is stiffer for an initial deformation cycle compared to subsequent cycles, even after several hours of rest (distinguishing it from a viscoelastic effect).^29^ As MS in our experiments regained effectiveness in causing 1:1 capture after a 1 min period of normal sinus rhythm, strain softening is unlikely to be a mechanism. More importantly, while strain softening is evident in non-viable (electrically inexcitable) myocardium, it is not observed in viable preparations undergoing physiologically relevant deformation, even in the presence of 2,3-butanedione monoxime (which inhibits cross-bridge formation, thus excluding an active mechanical component).^30^ In viable samples, there is, however, a *reversible* decrease of muscle stiffness that occurs between the first and subsequent deformation cycles, requiring ∼30 s of rest to recover. This viscoelastic effect could potentially account for a mechano-electric adaptation period in our (and others’) experiments, which is further supported by the observed stimulation rate-dependent decrease in the number of MS before a loss of capture. However, the fact that mixed MS:ES trains resulted in loss of VE_M_-induction with fewer MS than MS-only trains and at a *reduced* effective rate of MS, makes this explanation unlikely. Moreover, the fact that different MS:ES ratios ( ≤1:2 vs. ≥1:3) had different effects on the total number of stimulations and time to loss of MS capture, and the observation that changes in the time to loss of MS capture did not scale with the change in MS rate, seems to indicate that mediators of MS and ES are in fact (at least partially) overlapping (which would again make a viscoelastic effect a less likely mechanism).

There is evidence to support the idea that SAC_NS_ (or other ion channels) show ‘mechanical refractoriness’. For SAC_NS_, repetitive MS has been shown to cause a decrease in activated ion current. For instance, in acutely isolated embryonic chick heart cells, repeated MS causes a reduction in measured SAC_NS_ current, unless subsequent MS are spaced minutes apart.^31^ This use-dependent decrease in SAC_NS_ current could be responsible, in part at least, for the rate-dependent decrease in the number of MS to a loss of capture with repetitive MS seen in our experiments. At the same time, the ‘run-down’ of MS-inducible current is particularly pronounced after the first MS, with progressively less prominent reductions in SAC_NS_ current, which does not explain the faster loss of MS capture if interspersed with ES.

Mechanical effects on ionic concentrations, especially via modulation of sub-cellular compartments and/or sarcolemmal structures, may be another contributor to a loss of mechanical excitability. For instance, stretch has been shown to directly affect intracellular calcium handling in cardiac cells,^32^ including acute increases in localised sarcoplasmic reticulum calcium release events (calcium sparks) in ventricular myocytes,^33^ which reduces sarcoplasmic reticulum calcium levels. If alterations in calcium handling (such stretch-induced calcium release) are involved in VE_M_, then a depletion in sarcoplasmic reticulum calcium stores in general, or of a mechanically releasable sub-pool in particular, could affect MS effectiveness. An increase in calcium spark rate with stretch is thought to result either from direct mechanical stimulation of ryanodine receptor channels,^33^ or via effects mediated by reactive oxygen species,^34^ with microtubules acting as potential mechano-conductors. Both mechanisms could be affected by the frequency of cyclic MS, which could explain the stimulation rate-dependent decrease in the number of MS before a loss of capture in our experiments. Other sub-cellular compartments could include mechanically-induced calcium release from mitochondria (whose intra-organelle calcium concentrations may be similarly affected by stretch),^35^ or acidic stores (e.g. lysosomes).^36^ Equally, mechanical effects on membrane domains, such as stretch-induced incorporation of caveolae into the sarcolemma,^37^ have been reported and may play roles in electrical responses to MS.^38^^,^^39^

Why MS trains interspersed with ES should be accompanied by an accelerated loss of VE_M_-inducibility is not obvious, though, from any of the above candidate mechanisms.

### Future directions

**Figure 7:**
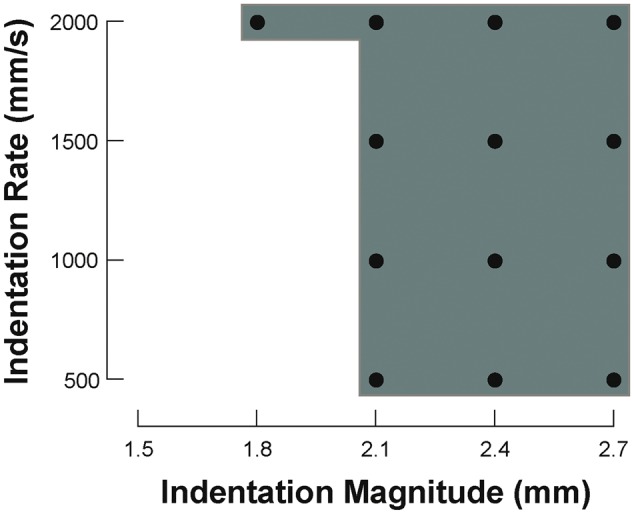
Inter-dependence of mechanically-induced excitation on indentation magnitude and rate. • indicates combinations of mechanical stimulation characteristics that elicited excitation.

Defining the mechanisms responsible for differences in the maximum rate and sustainability of pacing with MS compared to ES requires further study. To determine the relative contributions of potential mechanisms (altered ion channel activity, ionic concentrations, intracellular or sarcolemmal domains and tissue mechanics) experiments should involve: (i) pharmacological modulation of relevant ion fluxes and/or sub-cellular structures; (ii) domain-specific modulation or reporting of changes in ion concentrations and/or buffering; (iii) alteration of extracellular biophysical properties, from composition of solutions to background ventricular pressure/volume load or material stiffness (cytoskeletal disruption and cross-bridge inhibition); and (iv) variation of MS characteristics (indentation magnitude and rate, force and contact area/pressure under the probe), combined with measurement of strain and force (preliminary results have shown an inter-dependence of VE_M_ on indentation magnitude and rate, *Figure 7*) to assess, for example, whether MS at the point of failed 1:1 capture may still initiate VE_M_ if using higher mechanical stimulus intensities (‘relative refractoriness’). Additional key experiments involve work at various levels of structural integration, for example to determine maximum rates and sustainability of MS and ES in single isolated cardiomyocytes, or of the effects of non-myocytes^40^ on cardiomyocyte responses to MS and ES in cell culture^41^ or tissue slices,^42^ combined with quantitative computational integration of findings.^43^ Of further interest would be investigations into various pathophysiological states to determine the influence of disease background, and in multiple species, to test for conservation of effects. For applied research, it would be pertinent to determine, the lowest MS rates that allow sustenance of 1:1 capture, for instance during AV block, and to compare MS at multiple sites in the same heart, to assess ‘regionality’ of mechanical effects and to explore whether alternating MS between multiple sites could be an effective means of sustaining long-term mechanical pacing. This would also allow one to determine if, in contrast to the present results, previously-reported sustenance of MS in patients^5–8^^,^^11^ is related to differences in pacing rate (below vs. above sinus rate), experimental preparation (*in situ* vs. *ex situ*), species (human vs. rabbit), MS delivery (extracorporeal vs. epicardial), MS characteristics (such as magnitude and/or rate), and/or other factors.

## Conclusions

The use of MS for cardiac pacing at comparatively low rates in acutely asystolic hearts deserves consideration as a rapidly-applicable means for emergency heart rhythm management. Primary asystole/bradycardia can develop with cardiac arrest, atrioventricular block, or after electrical defibrillation, and mechanical pacing may be effective as a bridge to instrumentation-based approaches, in particular in out-of-hospital and emergency settings. This requires a thorough understanding of the mechanisms and limitations of MS. Here we show that the maximum rate at which MS can be used for same-site LV pacing is lower than that for ES, and that with MS overdrive pacing there is a rate-dependent decrease in the number of MS to a loss of capture. The mechanism(s) of differences in the ability of MS vs. ES to pace the heart are currently unknown, warranting further investigation.

## Supplementary material

Supplementary material is available at *Europace* online.

## Supplementary Material

Supplementary Data
