# Supplementary figures and images for: Comparing maximum rate and sustainability of pacing by mechanical vs. electrical stimulation in the Langendorff-perfused rabbit heart
Source: Europace. 2016 Dec 23;18(Suppl 4):iv85–93. doi: 10.1093/europace/euw354 (PMC5400084; doi:10.1093/europace/euw354)

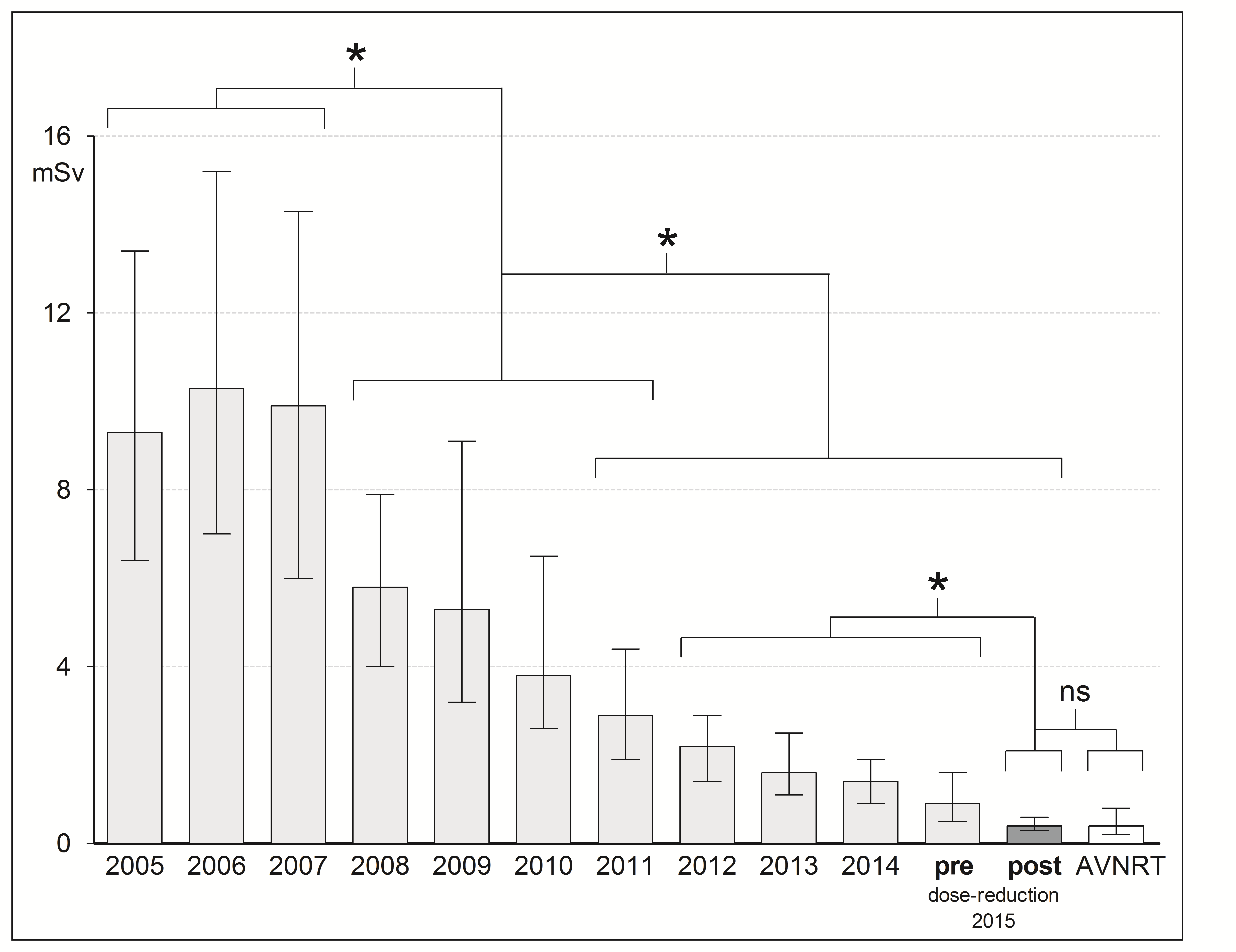

Supplement: Supplementary Data [file euw354_supp.png]
